# Free Fatty Acid Determination in Broccoli Tissues Using Liquid Chromatography–High-Resolution Mass Spectrometry

**DOI:** 10.3390/molecules29040754

**Published:** 2024-02-06

**Authors:** Christiana Mantzourani, Irene-Dimitra Mesimeri, Maroula G. Kokotou

**Affiliations:** Laboratory of Chemistry, Department of Food Science and Human Nutrition, Agricultural University of Athens, 11855 Athens, Greece; chrmantz@chem.uoa.gr (C.M.); irene.mesimeri@aua.gr (I.-D.M.)

**Keywords:** broccoli, free fatty acids, high-resolution mass spectrometry, hydroxy fatty acids, LC–HRMS, ricinoleic acid

## Abstract

Broccoli (*Brassica oleracea* L. var. *italica* Plenck) is a widely consumed vegetable, very popular due to its various nutritional and bioactive components. Since studies on the lipid components of broccoli have been limited so far, the aim of the present work was the study of free fatty acids (FFAs) present in different broccoli parts, aerial and underground. The direct determination of twenty-four FFAs in broccoli tissues (roots, leaves, and florets) was carried out, using a liquid chromatography–high-resolution mass spectrometry (LC-HRMS) method in a 10 min single run. Linolenic acid was found to be the most abundant FFA in all different broccoli parts in quantities ranging from 0.76 to 1.46 mg/g, followed by palmitic acid (0.17–0.22 mg/g) and linoleic acid (0.06–0.08 mg/g). To extend our knowledge on broccoli’s bioactive components, for the first time, the existence of bioactive oxidized fatty acids, namely hydroxy and oxo fatty acids, was explored in broccoli tissues adopting an HRMS-based lipidomics approach. 16- and 2-hydroxypalmitic acids were detected in all parts of broccoli studied, while ricinoleic acid was detected for the first time as a component of broccoli.

## 1. Introduction

Broccoli (*Brassica oleracea* L. var. *italica* Plenck), belonging to the *Brassicaceae* family, grows as an annual herb that is widely consumed as a vegetable around the world, with an ability for cultivation under a variety of agro-climatic conditions. A fully grown broccoli plant consists of several parts. The main head is composed of green clusters of flower buds (florets) arranged on longer stems branching out from a thicker stalk. Broccoli possesses shallow roots and leaves that are large and similar to other members of the cabbage family [1]. Broccoli sprouts also bear many small flower heads and are increasingly consumed worldwide [2]. Broccoli has become very popular due to its health benefits and various nutritional and bioactive components. It is a source of important minerals (e.g., potassium, calcium, and iron) as well as vitamins C, A, E, and K, while it is also a rich source of dietary fiber, amino acids, and antioxidants, such as polyphenolics and flavonoids (e.g., quercetin) [3,4].

To date, several reviews centering on the health benefits of broccoli and its bioactive constituents have been published [3,4,5,6]. Importantly, broccoli contains glucosinolates, secondary metabolites that produce bioactive isothiocyanates upon hydrolysis. Among isothiocyanates, sulforaphane has gained great attention due to its reported health-promoting properties, specifically anti-cancer, anti-inflammatory, immunomodulatory, antimicrobial, anti-diabetic, and cardio-protective effects, among others [7,8,9]. Additionally, broccoli is a rich source of n-3 fatty acids (FAs), with α-linolenic acid being the most predominant. This is an essential C18:3 n-3 polyunsaturated fatty acid (PUFA) that can be elongated to form other n-3 PUFAs, such as eicosapentaenoic acid (EPA). Such compounds exhibit diverse effects, such as anti-inflammatory or pro-resolution effects, either directly or through their oxylipin metabolites [10]. α-linolenic acid, in particular, exerts anti-inflammatory and immunomodulating effects, while its intake has been linked to a reduced risk of mortality from cardiovascular disease [11,12].

Lipids and, in particular, FAs in broccoli have not been extensively discussed in the literature, as they are minor broccoli constituents. In the existing reports, FAs are extracted from broccoli either through conventional solvent extraction [13,14], soxhlet extraction [15,16], or supercritical fluid extraction (SFE), which is a more environmentally friendly approach [17]. Generally, FAs are characterized as methyl esters (FAMES) using gas chromatography (GC) coupled to flame ionization (FID) [13,14,15,18], or mass spectrometry (MS) detectors [16,17], though UPLC-MS/MS was recently utilized for a metabolomics analysis of broccoli seeds and sprouts, including FAs [19]. In most cases, research studies focus on the FA composition of florets, sprouts, and seeds, while there have been limited reports regarding broccoli by-products, such as leaves, stems, and stalks, although they make up a considerable proportion of a whole broccoli plant. In these reports, it is demonstrated that broccoli leaves and stems contain similar quantities of PUFAs and other nutrients to those of broccoli florets, while roots are very rarely considered in studies [13,14,16,20].

Up to now, previous research has explored in broccoli the composition of FAs, which are present in their esterified form, while information about the presence of free fatty acids (FFAs) is missing. FFAs act directly on cell surface receptors (free fatty acid receptors FFA1, FFA2, FFA3, and FFA4) [21,22,23,24], which function as nutrient sensors. Long-chain FFAs, in particular n-3 PUFAs, such as α-linolenic acid, serve as ligands for FFA1 and FFA4, thus being able to affect energy homeostasis and contribute in controlling metabolic disorders [22,23,24]. The aim of our work was the study of a set of FFAs in different broccoli parts, aerial and underground, estimating the content of each particular FFA. Herein, we apply a liquid chromatography–high-resolution mass spectrometry (LC-HRMS) method for the direct determination of FFAs, following a simple extraction protocol for sample preparation and avoiding derivatization. Furthermore, in an effort to gain a better insight into the bioactive components of broccoli, we explore for the first time in broccoli the presence of bioactive oxidized FAs, namely hydroxy and oxo FAs.

## 2. Results and Discussion

### 2.1. Plant Material

In the present study, our attention was mainly focused on the FFA contents in roots, because roots constitute an underutilized part of broccoli rarely studied for bioactive components. Thus, root samples were collected from five different plants: four from conventionally cultivated plants and one from an organically cultivated broccoli plant. For comparison, floret samples from three different plants (two conventionally cultivated and one organically cultivated) and leaf samples from two plants (one organically cultivated and one conventionally cultivated) were included in the study.

### 2.2. LC-ESI-HRMS Data

The present LC-HRMS method allowed the simultaneous determination of twenty-four FAs, including medium- and long-chain saturated Fas, as well as monounsaturated fatty acids (MUFAs) and PUFAs, in broccoli samples. More specifically, the common FAs C6:0, C8:0, C9:0, C10:0, C12:0, C14:0, C14:1, C15:0, C16:0, C16:1, C17:0, C17:1, C18:0, C18:1 (oleic acid), C18:1 (petroselinic acid), C18:2, C18:3, C20:0, C20:3, C20:4, C20:5, C22:0, C22:5, and C22:6 were analyzed. The full list of FAs, together with their exact masses [M − H]^−^, their chromatographic retention times (t_R_), limits of detection (LOD), and quantification (LOQ), previously reported [25,26,27] regarding the analysis of milk, human plasma, and yogurt samples, are briefly summarized in Table S1 (Supplemental Data).

In addition, nine regio-isomers of hydroxypalmitic acid (2HPA, 3HPA, 6HPA, 7HPA, 8HPA, 9HPA, 10HPA, 11HPA, 16HPA), nine regio-isomers of hydroxystearic acid (2HSA, 3HSA, HSA, 7HSA, 8HSA, 9HSA, 10HSA, 11HSA, 12HSA), seven regio-isomers of oxopalmitic acid (14OPA, 10OPA, 9OPA, 8OPA, 7OPA, 6OPA, 5OPA), ten regio-isomers of oxostearic acid (16OSA, 12OSA, 10OSA, 9OSA, 8OSA, 7OSA, 6OSA, 5OSA, 4OSA, 3OSA), and ricinoleic acid were included in the study.

### 2.3. Analysis of Samples

**Figure 1:**
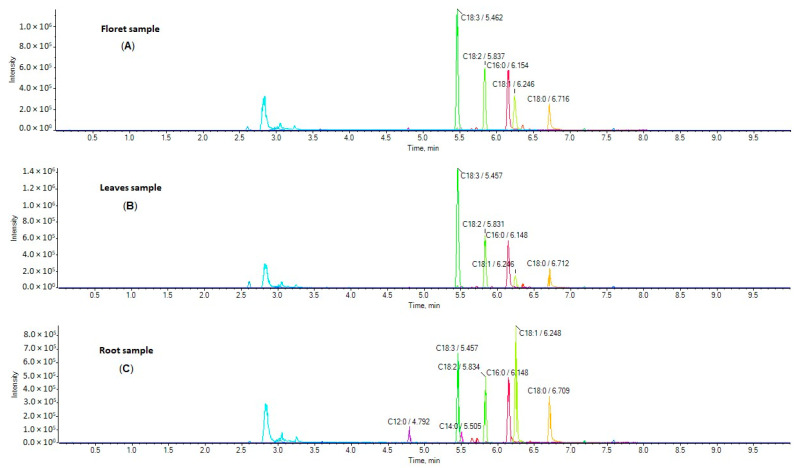
EICs of FFAs in a representative sample of broccoli florets (**A**), leaves (**B**), and roots (**C**).

Five broccoli root samples, three floret samples, and two leaf samples harvested from five different regions in Greece were analyzed. Representative extracted ion chromatograms (EICs) of a floret sample (A), leaf sample (B) and root sample (C) are shown in Figure 1. The simultaneous determination of 24 FAs was achieved in a single 10 min run. The contents of these analytes in broccoli samples (in triplicates) are summarized in Table 1, and they are expressed as mg of FA per g of dried broccoli sample.

In five root samples, linolenic acid was found to be the most predominant FA, in quantities ranging from 0.60 to 1.12 mg/g dried sample (Table 1). Interestingly, the highest quantity of linolenic acid in root samples was observed in a sample of broccoli fertilized with nitrogen sulfur and the lowest quantity in a sample of the organically cultivated broccoli. Additionally, other FAs that were found in high quantities in root samples were palmitic acid (0.19 to 0.27 mg/g), oleic acid (0.07 to 0.14 mg/g), linoleic acid (0.04 to 0.08 mg/g), stearic acid (0.07 to 0.10 mg/g), and myristic acid (0.03 to 0.07 mg/g). Eleven fatty acids were estimated at concentrations between 0.01 and 0.05 mg/g (C6:0, C8:0, C9:0, C10:0, C12:0, C15:0, C16:1, C17:0, C20:0, C20:3, C22:0) and the remaining FAs were not detected (Table 1).

In two leaf samples, linolenic acid was once more the most predominant FA in quantities between 1.24 and 1.68 mg/g dried sample, moderately higher than root samples. Subsequently, palmitic acid (0.17 to 0.24 mg/g), linoleic acid (0.04 to 0.11 mg/g), and stearic acid (0.06 to 0.06 mg/g) were found at higher concentrations. Notably, these major FAs were most abundant in leaves of the organically cultivated broccoli. Eleven FAs were found at concentrations ranging from 0.01 to 0.05 mg/g (C6:0, C8:0, C9:0, C10:0, C12:0, C14:0, C15:0, C17:0, C18:1, C20:0, C20:3) and nine FAs were not detected (Table 1).

Finally, in three floret samples, linolenic acid was again confirmed as the most abundant FA, at concentrations between 0.34 and 1.33 mg/g, lower than the corresponding leaf samples. Similar to leaf samples, palmitic acid (0.11 to 0.25 mg/g), linoleic acid (0.02 to 0.10 mg/g), and stearic acid (0.05 to 0.06 mg/g) were estimated at higher concentrations in floret samples and specifically in florets of the organically cultivated broccoli. Ten FAs were found at concentrations ranging from 0.01 to 0.06 mg/g (C6:0, C8:0, C9:0, C10:0, C12:0, C14:0, C15:0, C17:0, C18:1, C20:3) and the remaining FAs were not quantified (Table 1).

**Figure 2:**
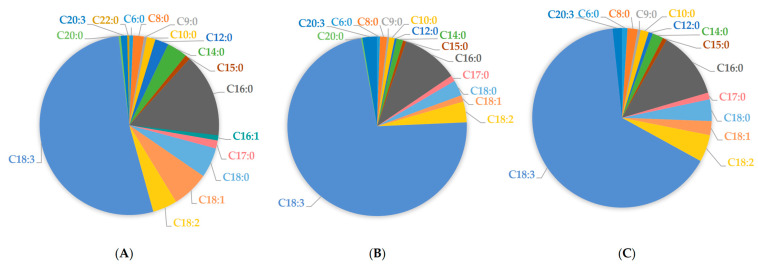
Free fatty acid average content in broccoli tissues: root samples (**A**), leaf samples (**B**), and floret samples (**C**).

Overall, in root samples tested herein, linolenic acid comprised 52.21% of total FFAs, linoleic acid 4.40%, oleic acid 6.66%, and palmitic acid 15.01%, respectively (Figure 2A). Accordingly, in leaf samples, linolenic acid comprised 72.35% of total FAs, linoleic acid 3.71%, palmitic acid 10.17%, oleic acid 1.16%, and stearic acid 2.80%, respectively (Figure 2B). In floret samples, linolenic acid comprised 64.94% of total FFAs, linoleic acid 4.85%, palmitic acid 12.08%, oleic acid 2.51%, and stearic acid 3.88%, respectively (Figure 2C).

To our knowledge, there are no existing reports regarding the determination of free (non-esterified) fatty acids in broccoli. In most cases, FAs in broccoli parts are presented as percentages of total FAs (free and esterified) after conversion to the corresponding FAMEs. Nonetheless, our findings generally follow the same trend as such reports regarding the abundance of different FAs. Specifically, in root samples, linolenic acid reportedly comprises 14.1–28.0% of total FAs, linoleic acid 9.2–14.9%, and oleic acid 26.4–36.2% of total FAs [13]. Furthermore, floret samples have been found to contain 3.2–45.4% linolenic acid, 15.4–18.7% linoleic acid, 15.8–34.3% palmitic acid, 4.1–6.2% oleic acid, and 1.3–10.4% stearic acid, respectively [14,15,16,18]. Finally, in leaf samples reported in literature, linolenic acid comprises 41.5–51.7% of total FAs, linoleic acid 12.6–14.8%, palmitic acid 23.7–31.2%, oleic acid 2.5–4.3%, and stearic acid 5.1–6.8%, respectively [14].

Based on our findings, broccoli roots as well as leaves contain significant amounts of FFAs, comparable to those of florets. Two of the most abundant FFAs in all the tested samples were linolenic acid and linoleic acid, corresponding to 56.6% of FFAs found in roots, 76.1% of FFAs found in leaves, and 69.8% of FFAs found in florets. These are essential (cannot be biosynthesized) PUFAs that are crucial for human health and nutrition, as briefly mentioned in the introduction. It has been demonstrated that higher dietary intake of ω-3 PUFAs, especially linolenic acid, is associated with a lower risk of mortality from cardiovascular and coronary heart disease [11,12] and a positive effect on obesity-related metabolic diseases [28], while linolenic acid has also displayed neuroprotective properties against stroke [29]. Therefore, the consumption of vegetables, fruits, other foods, and/or supplements rich in linolenic acid and other PUFAs can be highly beneficial. In the literature, considerable effort has been focused on the evaluation of vegetable by-products, like broccoli by-products, as sources of bioactive compounds and as functional food additives [30,31,32,33]. For instance, broccoli leaves have been previously utilized as leaf powder added in noodle products [34], gluten-free mini sponge cakes [35], and green tea [36]. The findings of the present study suggest that broccoli roots are worthy of attention for potential exploitation due to their linolenic and linoleic acid components.

Previously, our group has studied the existence of uncommon oxidized saturated FAs in dairy products [27,37,38]. Applying both suspect and targeted lipidomics approaches, we have identified in milk and yogurt previously unrecognized families of saturated hydroxy fatty acids (SHFAs) and saturated oxo fatty acids (SOFAs) with interesting biological activities [27,39]. Hydroxystearic acids (HSAs) and hydroxypalmitic acids (HPAs) exhibit anti-proliferative activity, while particular regio-isomers, 7HSAs and 9HSAs, protect β-cells from cytokine-induced apoptosis [33]. SOFAs, in particular 6OSA and 7OSA, were found to suppress the expression of both STAT3 and c-myc [40].

**Figure 3:**
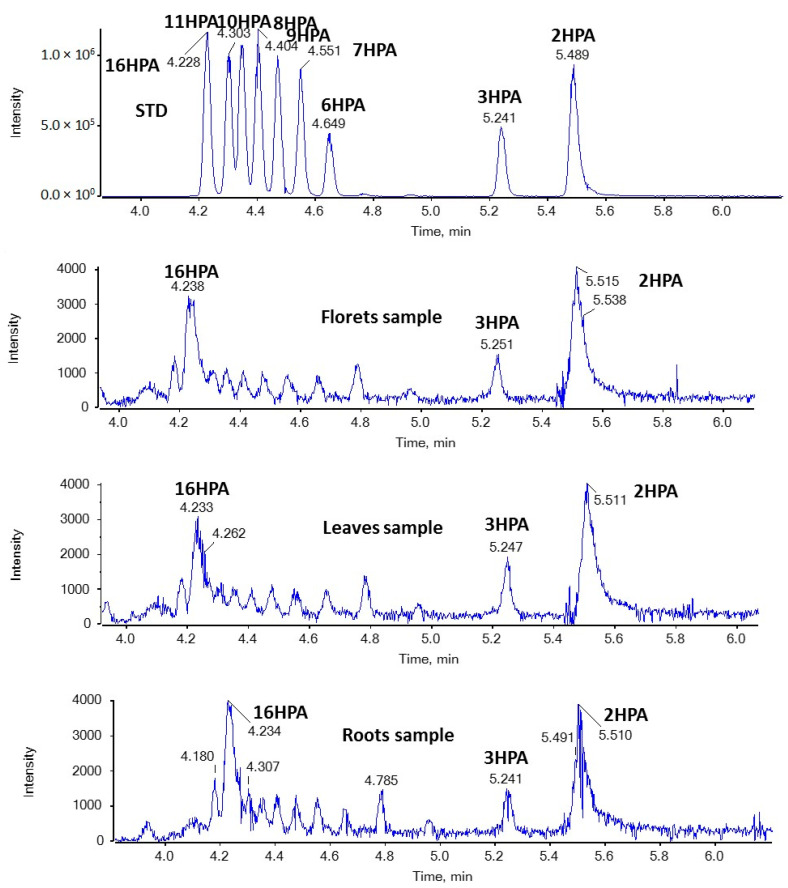
EICs of HPAs (*m*/*z* 271.2279) in a standard solution and representative samples of broccoli florets, leaves, and roots.

**Figure 4:**
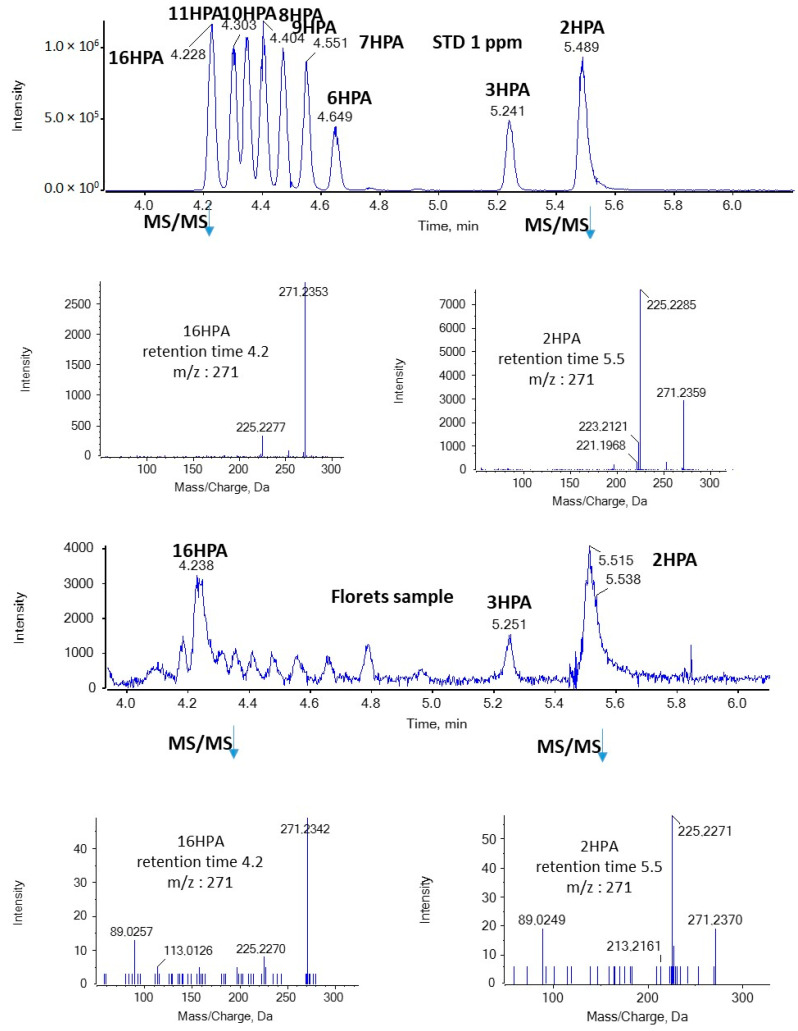
MS/MS spectra of 16HPA and 2HPA in a standard solution and a representative sample of broccoli florets.

In the present study, we were able to detect for the first time both SHFAs and SOFAs in broccoli samples. As shown in Figure 3, in the case of HPAs, the most intense peaks with *m*/*z* 271.2279 (corresponding to HPAs) were observed at 4.23 and 5.51 min in floret, leaf, and root samples. Based on the retention time, these peaks suggest the presence of 16HPA and 2HPA. In addition, the MS/MS spectra of the precursor ions [M − H]^−^ (*m*/*z* 271.2279) match those of standards for 16HPA and 2HPA, respectively, as depicted in Figure 4.

**Figure 5:**
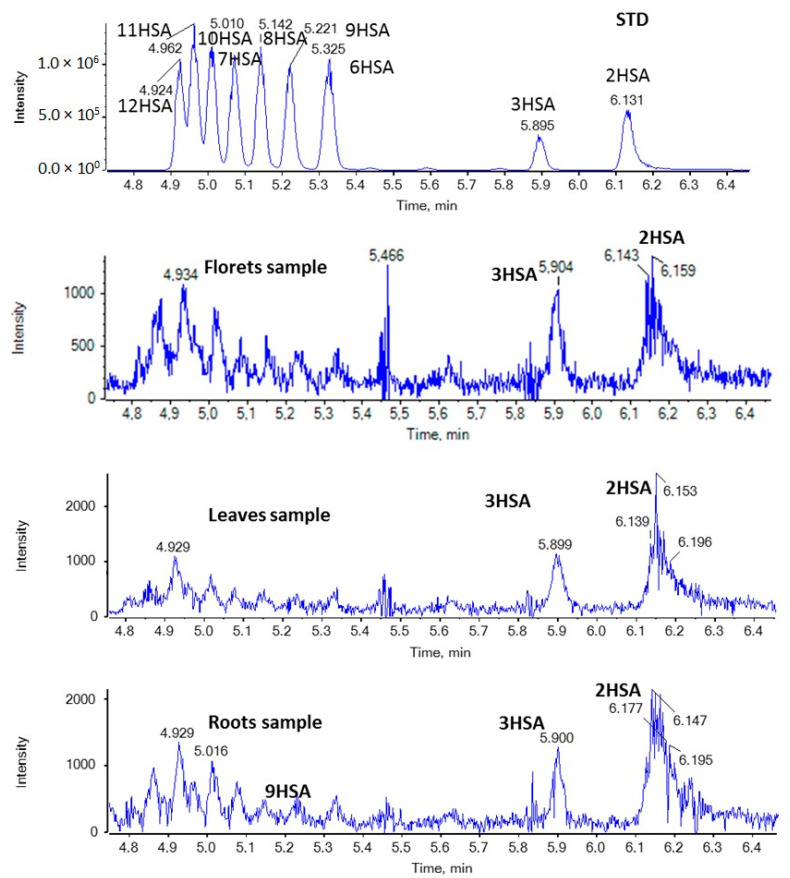
EICs of HSAs (*m*/*z* 299.2592) in a standard solution and representative samples of broccoli florets, leaves, and roots.

In the case of HSAs, as shown in Figure 5, 2HSA and 3HSA seem to be present in all broccoli samples in minor quantities.

**Figure 6:**
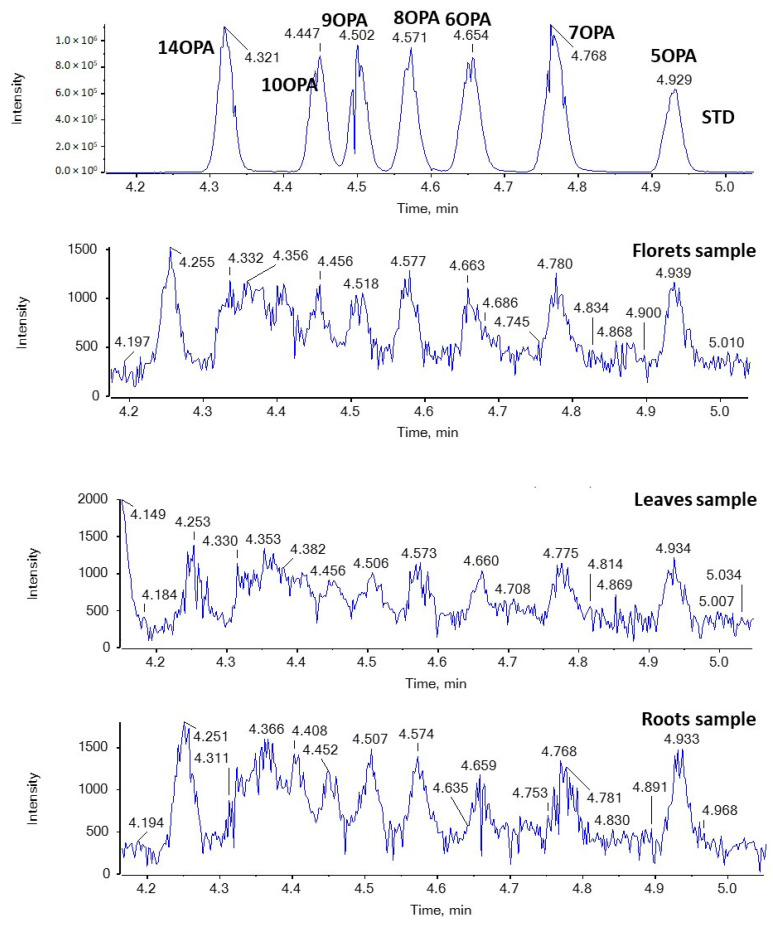
EICs of OPAs (*m*/*z* 269.2122) in a standard solution and representative broccoli floret, leaf, and root samples.

**Figure 7:**
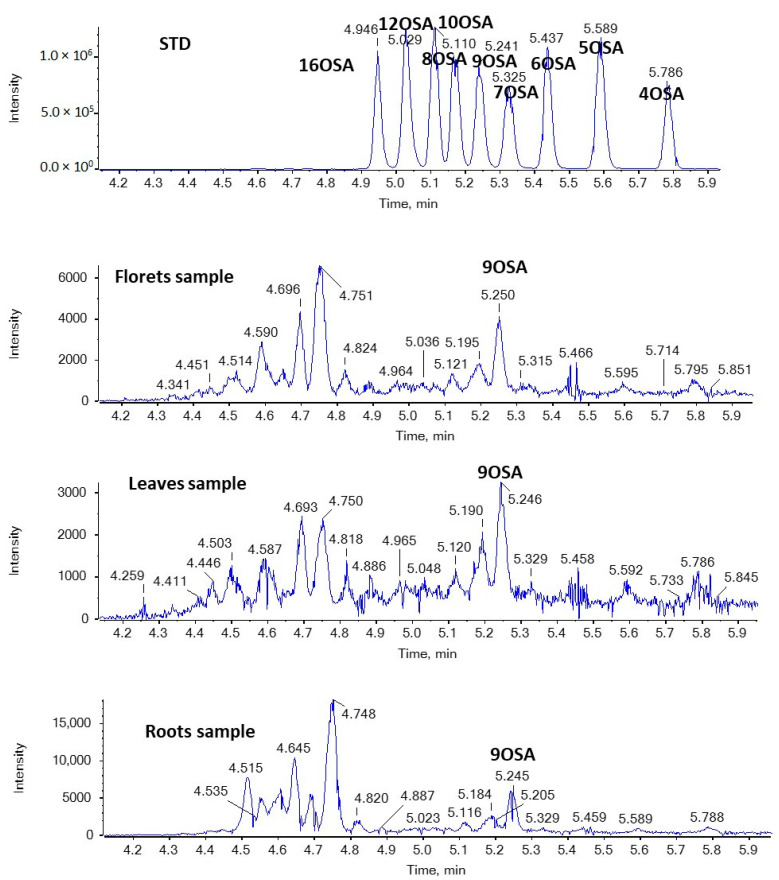
EICs of OSAs (*m*/*z* 297.2435) in a standard solution and representative broccoli floret, leaf, and root samples.

**Figure 8:**
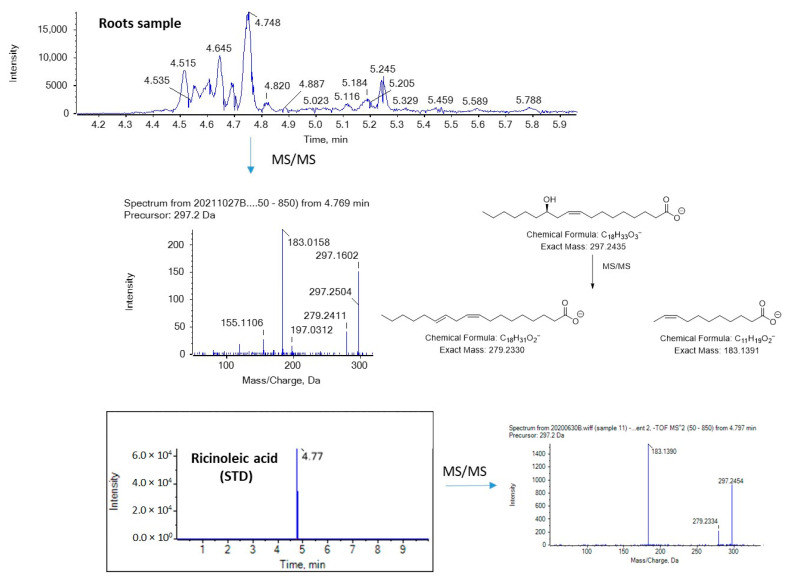
Mass spectrometry analysis of ricinoleic acid in a broccoli root sample.

Figure 6 and Figure 7 show EICs of OPAs and OSAs, respectively, in a standard solution and representative broccoli floret, leaf, and root samples. Regarding OSAs, 9OSA seems to be the most abundant (Figure 7). However, the most intense peak, with *m*/*z* 297.2435 (corresponding to OSAs), was observed at 4.75 min in floret and root samples. This peak was likely to correspond to ricinoleic acid (12-hydroxy-9-*cis*-octadecenoic acid), as we had previously detected this analyte in milk samples [38]. Indeed, in the MS/MS spectrum of the precursor ion [M − H]^−^ (*m*/*z* 297.2435), which corresponds to the analyte eluted at 4.75 min, two main fragments were observed at *m*/*z* 183.1390 and *m*/*z* 279.2328, corresponding to α-cleavage of ricinoleic acid and loss of H_2_O, respectively (Figure 8). This fragmentation matches that of a standard solution of ricinoleic acid, while the retention time for a reference sample of ricinoleic acid, under the same chromatographic conditions, was found to be 4.77 min (Figure 8).

## 3. Materials and Methods

### 3.1. Chemicals and Reagents

All the solvents used were of LC-MS analytical grade. Acetonitrile was purchased from Carlo Erba (Val De Reuil, France), isopropanol and methanol from Fisher Scientific (Laughborough, UK), and formic acid 98–100% from Chem-Lab (Zedelgem, Belgium). Caproic acid was purchased from Alfa Aesar (>98%, Lancaster, UK); caprylic acid, capric acid, myristic acid, myristoleic acid, pentadecanoic acid, margaric acid, linoleic acid, linolenic acid, arachidonic acid, and *cis*-4,7,10,13,16,19-docosahexaenoic acid from Sigma Aldrich (>99%, Steinheim, Germany); nonanoic acid, *cis*-10-heptadecenoic acid, arachidic acid, bihomo-γ-linolenic acid, *cis*-7,10,13,16,19-docosapentaenoic acid, 2-hydroxypalmitic acid (2HPA), and 2-hydroxystearic acid (2HSA) from Cayman Chemical Company (>98%, Ann Arbor, MI, USA); lauric acid from Acros Organics (>99%, Geel, Belgium); palmitic acid, 9-palmitoleic acid, stearic acid, oleic acid, petroselinic acid, behenic acid, and *cis*-5,8,11,14,17-eicosapentaenoic acid from Fluka (analytical standard, Karlsruhe, Germany); and 16-hydroxypalmitic acid (16HPA) from Sigma-Aldrich (Darmstadt, Germany). Also, 11-hydroxypalmitic acid (11HPA), 10-hydroxypalmitic acid (10HPA), 9-hydroxypalmitic acid (9HPA), 8-hydroxypalmitic acid (8HPA), 7-hydroxypalmitic acid (7HPA), 6-hydroxypalmitic acid (6HPA), 3-hydroxypalmitic acid (3HPA), 12-hydroxystearic acid (12HSA), 11-hydroxystearic acid (11HSA), 10-hydroxystearic acid (10HSA), 9-hydroxystearic acid (9HSA), 8-hydroxystearic acid (8HSA), 7-hydroxystearic acid (7HSA), 6-hydroxystearic acid (6HSA), and 3-hydroxystearic acid (3HSA) were synthesized as previously described [39] and provided by Prof. G. Kokotos. Finally, 14-oxopalmitic acid (14OPA), 10-oxopalmitic acid (10OPA), 9-oxopalmitic acid (9OPA), 8-oxopalmitic acid (8OPA), 7-oxopalmitic acid (7OPA), 6-oxopalmitic acid (6OPA), 5-oxopalmitic acid (5OPA), 16-oxostearic acid (16OSA), 12-oxostearic acid (12OSA), 10-oxostearic acid (10OSA), 9-oxostearic acid (9OSA), 8-oxostearic acid (8OSA), 7-oxostearic acid (7OSA), 6-oxostearic acid (6OSA), 5-oxostearic acid (5OSA), and 4-oxostearic acid (4OSA) were synthesized as previously described [40] and provided by Prof. G. Kokotos.

### 3.2. Stock and Working Solutions

Stock solutions of the standard compounds (1000 mg/L in methanol) were prepared and stored at 4 °C. Working standard solutions (500 ng/mL) were prepared daily by appropriate dilution.

### 3.3. Instrumentation

Chromatography was performed on a Halo C18 column (2.7 μm, 90 Å, 0.5 × 50 mm) from Eksigent, using a micro-LC Eksigent (Eksigent, Darmstadt, Germany) equipped with an autosampler set at 5 °C and a thermostated column compartment. Separation was performed with a gradient over the course of 10 min at a flow rate of 55 µL/min, using a mobile phase system consisting of solvent A: H_2_O/0.01% and solvent B: acetonitrile/0.01% formic acid/isopropanol 80/20 *v*/*v*. The gradient elution program was as follows: 0–0.5 min, 5% B; 0.5–8.0 min, gradually increasing to 98% B; 8.0–8.5 min, 98% B, followed by a 1.5 min equilibration step to the initial conditions prior to the next injection. The injection volume was set at 5 µL.

An ABSciex Triple TOF 4600 (ABSciex, Darmstadt, Germany) was used to perform the HRMS measurements and all the experiments were carried out by electrospray ionization (ESI) in negative mode. The data acquisition method consisted of a TOF-MS full scan *m*/*z* 50–850 and several information-dependent acquisition (IDA)-TOF-MS/MS product ion scans, using a 40 V collision energy (CE), with a 15 V collision energy spread (CES) used for each candidate ion in each data acquisition cycle (1091). The MS resolution working conditions were as follows: ion energy 1 (IE1) −2.3, vertical steering (VS1) −0.65, horizontal steering (HST) 1.15, and vertical steering 2 (VS2) 0.00. MultiQuant 3.0.2 and PeakView 2.1 (ABSciex, Darmstadt, Germany) were employed for the data acquisition. EICs were obtained creating the base peak chromatograms for masses that achieve a 0.01 Da mass accuracy width. The relative tolerance of the retention time was set within a margin of ±2.5%. The integration of the peak areas was performed manually using MultiQuant 3.0.2.

### 3.4. Sample Preparation

The sample preparation was carried out as previously described by Kokotou et al. [41], with some modifications. Briefly, 20 mL of McIIvaine buffer [0.2 M Na_2_HPO_4_ (16.47 mL), 0.1 M citric acid (3.53 mL)] (pH 7.0) was added into 1 g of dry broccoli tissue and the mixture was incubated in a water bath for 3 h at 45 ± 3 °C. After the addition of 30 mL CH_2_Cl_2_, the mixture was stirred for 15 min and then filtered using a Buchner funnel equipped with Whatman filter paper grade 1. The solid residue was extracted twice with 40 mL CH_2_Cl_2_. The extracts were dried with 1 g of anhydrous sodium sulfate and the solvent was then evaporated to dryness at 35 °C under vacuum, on a rotary evaporator. The residue was dissolved in 1 mL MeOH for LC-HRMS analysis.

### 3.5. Sampling

Roots were collected from five different *Brassica oleracea* L. var. *italica* Plenck plants as specified below: three conventionally cultivated non-fertilized plants from Agrinio (38°37′47″ N 21°24′42″ E) cultivar Parthenon, Vassilika (38°58′2″ N 23°21′23″ E) cultivar Marathon, and Vonitsa (38°54′59.00″ N 20° 53′11.00″ E) cultivar Marathon; one conventionally cultivated plant fertilized with nitrogen sulfur from Argos (37°39′38″ N 22°42′19″ E) cultivar Marathon; one organically cultivated plant from the Agricultural University of Athens field (37°59′2″ N 23°42′19″ E) cultivar Monrello, grown until florets were ready for human consumption. Florets were collected from three different *Brassica oleracea* L. var. *italica* Plenck plants: one conventionally cultivated non-fertilized plant from Vonitsa (38°54′59.00″ N 20°53′11.00″ E) cultivar Marathon; one conventionally cultivated non-fertilized plant from Athyra (40°49′44.7″ N 22°35′37.8″ E) cultivar Parthenon; one organically cultivated plant from the Agricultural University of Athens field (37°59′2″ N 23°42′19″ E) cultivar Monrello. Leaves were collected from two different *Brassica oleracea* L. var. *italica* Plenck plants: one conventionally cultivated non-fertilized plant from Vonitsa (38°54′59.00″ N 20° 53′11.00″ E) cultivar Marathon; one organically cultivated plant from the Agricultural University of Athens field (37°59′2″ N 23°42′19″ E) cultivar Monrello. All samples were lyophilized, and after grounding to a fine homogenous powder, stored at −20 °C.

### 3.6. Data Processing and Quantification

All chemical structures were drawn using ChemBioDraw Ultra 12.0 (PerkinElmer Informatics, Waltham, MA, USA). The data acquisition was carried out with MultiQuant 3.0.2 and PeakView 2.1 from (ABSciex, Darmstadt, Germany).

### 3.7. Method Validation

Broccoli samples were spiked with a mixed standard solution of all analytes at 500 ng/mL to estimate the recovery (%R), the relative standard deviation (%RSD), and the matrix factor (MF). As shown in Table S2 (Supplemental Material), satisfactory recoveries indicate the accuracy of the proposed method, while the precision was investigated by means of %RSD. The matrix factor was calculated as the ratio of the peak response in the presence of a matrix to the peak response in the pure solvent. Matrix factor values < 1 and >1 denote signal suppression and signal enhancement, respectively.

## 4. Conclusions

In conclusion, a set of twenty-four FFAs was studied in different broccoli parts (roots, leaves, and florets), estimating the content of each particular FFA, including medium- and long-chain saturated FAs, as well as MUFAs and PUFAs. The present method employs a simple extraction protocol without a derivatization step, allowing the direct determination of FFAs in a 10 min single run. Among the FAs that were estimated, linolenic acid was the most abundant FA in all different broccoli parts in quantities ranging from 0.76 to 1.46 mg/g, followed by palmitic acid (0.17–0.22 mg/g) and linoleic acid (0.06–0.08 mg/g). These results suggest that broccoli roots as well as leaves, which are underutilized broccoli by-products, could be a potential source of health-promoting FAs, such as linolenic acid, and could be utilized as additives or supplements in order to be incorporated in the human diet. Furthermore, an HRMS-based lipidomics approach exploring bioactive oxidized FAs was conducted for the first time in broccoli samples and revealed the presence of 16- and 2-hydroxypalmitic acids, as well as ricinoleic acid, in all parts of broccoli studied. The presence of such components may contribute to the bioactive properties of broccoli and broccoli by-products. Further studies are needed in future to enrich our knowledge on the existence of bioactive lipids in broccoli and to showcase the potential of exploitation of the underutilized broccoli parts due to their bioactive components.

## Figures and Tables

**Table 1:** Contents of free fatty acids in broccoli samples (mg/g).

	Root Samples (*n* = 5), Triplicates					Leaf Samples (*n* = 2), Triplicates			Floret Samples (*n* = 3), Triplicates				
Free Fatty Acid	Minimum Value (mg/g)	Maximum Value (mg/g)	Mean Value ± SD (mg/g)	%	Minimum Value (mg/g)	Maximum Value (mg/g)	Mean Value ± SD (mg/g)	%	Minimum Value (mg/g)	Maximum Value (mg/g)	Mean Value ± SD (mg/g)	%	
C6:0	0.01	0.01	0.01 ± 0.001	0.8	0.01	0.02	0.01 ± 0.001	0.5	0.01	0.02	0.01 ± 0.001	1.0	
C8:0	0.02	0.03	0.03 ± 0.001	1.9	0.03	0.03	0.03 ± 0.001	1.4	0.02	0.03	0.03 ± 0.001	1.9	
C9:0	0.00	0.01	0.01 ± 0.000	0.5	0.01	0.01	0.01 ± 0.001	0.4	0.01	0.01	0.01 ± 0.001	0.5	
C10:0	0.01	0.03	0.02 ± 0.001	1.5	0.02	0.02	0.02 ± 0.001	1.0	0.02	0.03	0.02 ± 0.001	1.5	
C12:0	0.01	0.05	0.04 ± 0.002	2.4	0.01	0.01	0.01 ± 0.001	0.5	0.01	0.01	0.01 ± 0.001	0.9	
C14:0	0.03	0.07	0.05 ± 0.003	3. 7	0.02	0.03	0.02 ± 0.001	1.2	0.02	0.03	0.03 ± 0.001	2.0	
C14:1	-	-	-	-	-	-	-	-	-	-	-	-	
C15:0	0.01	0.02	0.01 ± 0.001	1.0	0.01	0.01	0.01 ± 0.001	0.6	0.01	0.01	0.01 ± 0.001	0.8	
C16:0	0.19	0.27	0.22 ± 0.018	15.0	0.17	0.24	0.21 ± 0.009	10.2	0.11	0.25	0.17 ± 0.011	12.1	
C16:1	0.01	0.02	0.01 ± 0.001	0.9	-	-	-	-	-	-	-	-	
C17:0	0.02	0.03	0.02 ± 0.001	1.5	0.02	0.02	0.02 ± 0.001	1.1	0.02	0.02	0.02 ± 0.001	1.2	
C17:1	-	-	-	-	-	-	-	-	-	-	-	-	
C18:0	0.07	0.10	0.08 ± 0.003	5.4	0.06	0.06	0.06 ± 0.001	2.8	0.05	0.06	0.05 ± 0.003	3.9	
C18:1 Oleic acid	0.07	0.14	0.10 ± 0.006	6.7	0.02	0.03	0.02 ± 0.001	1.2	0.01	0.06	0.04 ± 0.002	2.6	
C18:1 Petroselinic acid	-	-	-	-	-	-	-	-	-	-	-	-	
C18:2	0.04	0.08	0.06 ± 0.004	4.5	0.04	0.11	0.08 ± 0.001	3.8	0.02	0.10	0.07 ± 0.001	4.9	
C18:3	0.60	1.12	0.76 ± 0.032	52.2	1.24	1.68	1.46 ± 0.052	72.4	0.34	1.33	0.92 ± 0.047	64.94	
C20:0	0.01	0.01	0.01 ± 0.001	0.4	0.01	0.01	0.01 ± 0.001	0.3	-	-	-	-	
C20:3 Bishomo-γ-linolenic	0.01	0.02	0.02 ± 0.001	1.2	0.01	0.10	0.05 ± 0.003	2.6	0.01	0.04	0.02 ± 0.001	1.8	
C20:4	-	-	-	-	-	-	-	-	-	-	-	-	
C20:5	-	-	-	-	-	-	-	-	-	-	-	-	
C22:0	0.01	0.01	0.01 ± 0.001	0.4	-	-	-	-	-	-	-	-	
C22:5	-	-	-	-	-	-	-	-	-	-	-	-	
C22:6	-	-	-	-	-	-	-	-	-	-	-	-	

SD: standard deviation.

## Data Availability

All data supporting this study are included in the article and Supplementary Materials.

## Supplementary Materials

The following supporting information can be downloaded at: https://www.mdpi.com/article/10.3390/molecules29040754/s1: Table S1: List of FAs, together with their exact masses [M − H]^−^, their chromatographic retention times (R_t_), limits of detection (LOD) and quantification (LOQ), Table S2: Accuracy (recovery%), precision data (RSD%) and matrix factor (MF) in spiked broccoli samples, Figure S1: Extracted ion chromatograms (EICs) of common FFAs in a representative floret sample.
