# Supplementary material for: Free Fatty Acid Determination in Broccoli Tissues Using Liquid Chromatography–High-Resolution Mass Spectrometry
Source: Molecules. 2024 Feb 6;29(4):754. doi: 10.3390/molecules29040754 (PMC10891939; doi:10.3390/molecules29040754)
Supplement: Supplementary file 1 [file molecules-29-00754-s001.zip › molecules-2814963-supplementary.pdf]

Supplementary Material

Free Fatty Acids Determination in Broccoli Tissues using Liquid Chromatography-High Resolution Mass Spectrometry

Christiana Mantzourani, Irene Mesimeri and Maroula G. Kokotou *

Laboratory of Chemistry, Department of Food Science and Human Nutrition, Agricultural University of Athens, Iera Odos 75, Athens 11855, Greece

*Corresponding author: Assist. Prof. Maroula G. Kokotou, Department of Food Science and Human Nutrition, Agricultural University of Athens, Iera Odos 75, Athens 11855, Greece, email: mkokotou@aua.gr

Table of contents

1. Table S1. List of analytes together with their exact masses $[M-H]^-$, their retention time R_t (min), and their limits of detection (LOD) and quantification (LOQ).

2. Table S2. Accuracy (recovery %), precision data (RSD %) and matrix factor (MF) in spiked broccoli samples.

3. Figure S1. Extracted ion chromatograms (EICs) of common FFAs in a representative floret sample.

Table S1. List of analytes together with their exact masses $[M-H]^-$, their retention time R_t (min), and their limits of detection (LOD) and quantification (LOQ).

Analyte	$[M-H]^-$	R_t (min)	LOD (ng/mL)	LOQ (ng/mL)
Caproic acid (C6:0) [1]	115.0765	2.1	0.5	1.5
Caprylic acid (C8:0) [1]	143.1078	3.3	0.5	1.1
Nonanoic acid (C9:0)[2]	157.1234	3.7	0.5	1.2
Capric acid (C10:0) [1]	171.1391	4.1	0.5	1.7
Lauric acid (C12:0) [1]	199.1704	4.9	0.6	1.8
Myristic acid (C14:0) [1]	227.2017	5.6	0.6	1.8
Myristoleic acid (C14:1 n-5) [1]	225.1850	5.1	0.6	1.8
Pentadecanoic acid (C15:0) [1]	241.2173	6.0	0.8	2.4
Palmitic acid (C16:0) [1]	255.2330	6.3	0.9	2.3
cis-9-Palmitoleic acid (C16:1 n-7) [1]	253.2173	5.8	1.6	4.8
Margaric acid (C17:0) [1]	269.2486	6.6	0.8	2.4
cis-10-Heptadecenoic acid (C17:1 n-7) [1]	267.2330	6.2	0.8	2.4
Stearic acid (C18:0) [1]	283.2643	6.8	0.9	2.8
Oleic acid (C18:1 n-9) [1]	281.2486	6.4	0.7	2.3
Petroselinic acid (18:1 n-12)	281.2486	6.3	0.6	2.1
Linoleic acid (C18:2 n-6) [1]	279.2330	6.0	0.6	1.8
total-Linolenic acid (C18:3)[1]	277.2173	5.5	0.6	1.8
Arachidic acid (C20:0) [1]	311.2956	7.0	0.8	2.4
Dihomo-γ-linolenic acid (C20:3 n-6) [1]	305.2486	6.1	0.6	1.8
Arachidonic acid (C20:4 n-6) [1]	303.2330	5.9	0.6	1.8
cis-5,8,11,14,17-Eicosapentaenoic acid (C20:5 n-3) [1]	301.2173	5.6	0.6	1.8
Behenic acid (C22:0)[2]	339.3269	7.7	0.8	2.4
cis-7,10,13,16,19-Docosapentaenoic acid (C22:5 n-3) [1]	329.2486	6.1	0.4	1.2
cis-4,7,10,13,16,19-Docosahexaenoic acid (C22:6 n-3) [1]	327.2330	5.9	0.4	1.2

References

1. Kokotou, M. G., Mantzourani, C., Kokotos, G. (2020). Development of a liquid chromatography-high resolution mass spectrometry method for the determination of free fatty acids in milk. *Molecules*, *25*, 1548.
2. Mantzourani, C., Batsika, C. S, Kokotou, M. G., Kokotos, G. (2022). Free fatty acid profiling of Greek yogurt by liquid chromatography-high resolution mass spectrometry (LC-HRMS) analysis. *Food Research International*, *160*, 111751.

Table S2. Accuracy (recovery %), precision data (RSD %) and matrix factor (MF) in spiked broccoli samples.

Analyte	Spike level 500 ng/mL		
	Recovery (%R)	RSD (%)	Matrix factor (MF)
C6:0	89	0.34	0.95
C8:0	125	1.96	0.78
C9:0	111	12.48	0.76
C10:0	100	2.12	0.94
C12:0	95	7.42	1.31
C14:0	115	1.73	1.32
C14:1	105	6.20	1.16
C15:0	119	13.09	1.02
C16:0	103	4.06	1.08
C16:1	103	1.76	1.33
C17:0	87	15.68	1.07
C17:1	92	11.08	1.24
C18:0	86	9.51	0.93
C18:1	83	5.82	1.39
C18:2	87	8.94	1.24
C18:3	79	11.52	1.40
C20:0	92	6.06	0.88
C20:3	97	6.01	0.90
C20:4	91	5.87	1.11
C20:5	89	2.50	0.80
C22:0	98	2.32	1.33
C22:5	89	3.26	1.21
C22:6	94	13.74	0.53

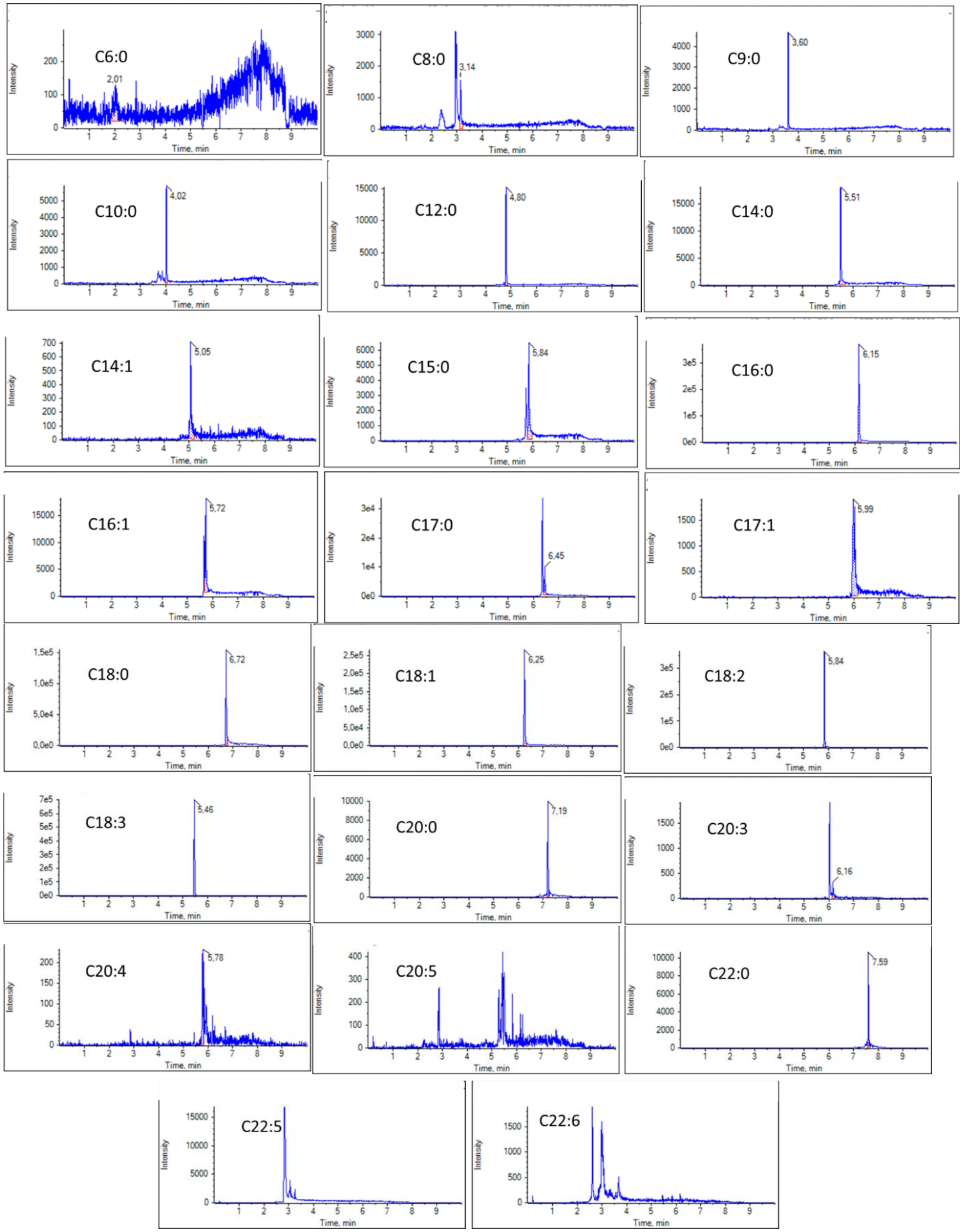

Figure S1. Extracted ion chromatograms (EICs) of common FFAs in a representative floret sample.
